# Development and Successful Validation of Simple and Fast TLC Spot Tests for Determination of Kryptofix^®^ 2.2.2 and Tetrabutylammonium in ^18^F-Labeled Radiopharmaceuticals

**DOI:** 10.3390/ph7050621

**Published:** 2014-05-14

**Authors:** Matthias Kuntzsch, Denis Lamparter, Nils Brüggener, Marco Müller, Gabriele J. Kienzle, Gerald Reischl

**Affiliations:** 1 Department of Preclinical Imaging and Radiopharmacy, Eberhard Karls University Tübingen, Röntgenweg 15, Tübingen D-72076, Germany; E-Mails: matthias.kuntzsch@med.uni-tuebingen.de (M.K.); lamparter_d@ukw.de (D.L.); nils.brueggener@med.uni-tuebingen.de (N.B.); gabriele.kienzle@med.uni-tuebingen.de (G.J.K.); 2 Department of Radiochemistry, ABX GmbH, Heinrich-Gläser-Straße 10, Radeberg D-01454, Germany; E-Mail: marco.mueller@abx.de

**Keywords:** Kryptofix^®^ 2.2.2, tetrabutylammonium, fluorine-18, quality control, thin layer chromatography (TLC) spot test

## Abstract

Kryptofix^®^ 2.2.2 (Kry) or tetrabutylammonium (TBA) are commonly used as phase transfer catalysts in ^18^F-radiopharmaceutical productions for positron emission tomography (PET). Due to their toxicity, quality control has to be performed before administration of the tracer to assure that limit concentration of residual reagent is not reached. Here, we describe the successful development and pharmaceutical validation (for specificity, accuracy and detection limit) of a simplified color spot test on TLC plates. We were able to prove its applicability as a general, time and resources saving, easy to handle and reliable method in daily routine analyzing ^18^F-tracer formulations for Kry (in [^18^F]FDG or [^18^F]FECh) or TBA contaminations (in [^18^F]FLT) with special regard to complex matrix compositions.

## 1. Introduction

Production of ^18^F-radiopharmaceuticals is most commonly done by labeling the respective precursor with [^18^F]fluoride via nucleophilic substitution. [^18^F]Fluoride is a cyclotron produced radionuclide (nuclear reaction: ^18^O(p,n)^18^F) from [^18^O]water. To assure reactivity of the [^18^F]fluoride, it is regularly dried azeotropically and phase transfer catalysts need to be added to enhance nucleophilicity of the anion. Most prominent example for this approach is the synthesis of 2-deoxy-2-[^18^F]fluoro-*d*-glucose ([^18^F]FDG) introduced by Hamacher *et al.* [1].

Kryptofix^®^ 2.2.2 (Kry), a crown ether, is one of the most widely used among the phase transfer reagents, another one is tetrabutylammonium (TBA; added e.g., as hydroxide, carbonate or hydrogen carbonate). Radiopharmaceutical formulations have to be analyzed for residual contaminations with these reagents before human application, due to their toxicity (e.g., Kry: LD_50_ (i.v.) in rodents = 32–35 mg/kg [2]). For Kry, limit in European Pharmacopoeia (Ph. Eur.) is 2.2 mg/V (*i.e.*, patient dose; see monograph for [^18^F]FDG {#1325; fludeoxyglucose (^18^F) injection} and other ^18^F-radiopharmaceuticals: [^18^F]FLT {#2460; 3'-deoxy-3'-[^18^F]fluorothymidine}, [^18^F]FMISO {#2459; [^18^F]fluoromisonidazole} [3]), in US Pharmacopoeia (USP) limit is <50 µg/mL [4]. For TBA in ^18^F-radiopharmaceuticals a limit of 2.6 mg/V is defined in Ph. Eur. (see same monographs as for Kry). Ph. Eur. suggests a color spot test on a thin layer chromatography (TLC) plate for Kry and a HPLC method for TBA in ^18^F-radiopharmaceutical monographs (see above).

In the past, various procedures have been described in the literature for identification and quantification of Kry, with the aim of providing specific and fast methods. TLC procedures are the most widespread, as they do not require sophisticated instrumental equipment. A color spot test on a TLC plate as used in monographs with iodoplatinate [5] for staining was described by Mock *et al.* [6]. To increase specificity and avoid false positive (e.g., from other amines) or false negative results (from stabilizers) thin layer chromatography systems were developed [7,8,9,10]. But also gas chromatography (GC; [11]), high-performance liquid chromatography (HPLC; [12]) or liquid chromatography-tandem mass spectrometry (LC/MS/MS; [13]) and even NMR or IR spectroscopy [14] were applied. More recently, a very fast (<1 min) and highly sensitive (lower limit = 0.5 ng/mL) rapid-resolution liquid chromatography MS/MS coupled system for analysis of Kry was published [15]. Still, these methods suffer from certain disadvantages. The latter (GC, LC, MS, NMR, IR) need high-quality instrumental equipment combined with a higher effort for validation. On the other hand, TLC tests are more time consuming due to development of plates or require the expensive reagent iodoplatinate. In addition, in our opinion to prove that in a certain radiopharmaceutical formulation the Kry (or TBA) content is below a defined limit—without the necessity of exact quantification, the test should be as simple and quick as possible, but nevertheless meeting pharmaceutical requirements. This particularly holds true in view of the fact that all literature data agree that, when synthesis and purification were performed successfully, Kry or TBA residues were extremely low and far from limits. When any test for Kry (or TBA) is applied to a newly produced radiopharmaceutical, it will always need some kind of validation, no matter how specific the test proved to be so far. Matrix effects may play an important role, pH as well. The important factor is how much time and effort it will take in daily routine.

After first promising results with the TLC spot tests as will be described below for Kry and TBA, we set up validations regarding specificity, accuracy and detection limit for [^18^F]FDG (Kry) and, to demonstrate a more general applicability, for 2-[^18^F]fluoroethylcholine ([^18^F]FECh; also Kry), where residual *N*,*N*-dimethylaminoethanol (DMAE) may interfere [16]. As the HPLC procedure for TBA analysis described in the monographs appeared to be challenging, the spot test was also to be validated for this reagent, with [^18^F]FLT as example [17]. As an additional important parameter, the stability of the necessary standard solutions containing the limit concentrations of Kry or TBA should also be investigated.

## 2. Experimental

### 2.1. General

All reagents were of highest purity available or of pharmaceutical grade, where necessary (article numbers in brackets) and were used as received. From Merck (Darmstadt, Germany) were purchased: Ethanol (1.00983), hydrochloric acid (1.09137), iodine (1.04761), Kryptofix 2.2.2 (8.14925), methanol (1.06007), NaCl (1.06404), NaH_2_PO_4_**^.^**2H_2_O (1.06345), NaOH (1.06482), NH_4_OH (25% in water; 1.05432) and trisodium-citrate (1.06448). TLC silica plates were from Macherey-Nagel (Düren, Germany, 805021), water for injection (WFI) was from Fresenius Kabi (Bad Homburg, Germany, 1636065), disodium-hydrogencitrate (35,908-4) and DMAE (391263) from Aldrich (Steinheim, Germany) and tetrabutylammonium hydroxide 30-hydrate from Sigma (Steinheim, Germany, 86859). Sterile phosphate buffered saline (PBS) was provided from the University pharmacy.

### 2.2. Syntheses of [^18^F]FDG, [^18^F]FECh and [^18^F]FLT

N.c.a. [^18^F]fluoride was produced at the PETtrace cyclotron (GE Healthcare, Uppsala, Sweden), via the ^18^O(p,n)^18^F nuclear reaction by irradiating 2.5 mL of >95% enriched [^18^O]water (Rotem, Be’er Sheva, Israel) with 16.5 MeV protons in a niobium target body with Havar^®^ foils. Products from automated syntheses were used as samples in the described validations. [^18^F]FDG was produced on a TRACERlab MX synthesizer (GE Healthcare), cassettes were from Rotem (Israel) and reagent kits from ABX (Radeberg, Germany) with the standard method supplied from GE except that the final volume was increased to 20 mL by addition of water for injection and 90 µL of ethanol for stabilization. The final matrix of the [^18^F]FDG formulation was a citrate buffer (specification limits pH 5.0–8.0; normally a pH value of 5.5–6.0 is found) containing a specified amount of ethanol with limits of 2000–5000 mg/L. [^18^F]FECh was produced on a modified TRACERlab FX F_N_ synthesizer (GE Healthcare) yielding 13 mL of [^18^F]FECh in a PBS buffer (pH 7.4). Synthesis of [^18^F]FLT was performed on TRACERlab MX with cassettes, synthesis software sequence and reagent kits from ABX. Here, the final formulation was again a citrate buffer (21 mL; nominal pH 5.0), containing 10% of ethanol.

### 2.3. Solutions for the Validation of Kryptofix 2.2.2 in [^18^F]FDG

(a) Matrix buffers: The buffers contained salt concentrations as in [^18^F]FDG formulations, *i.e.*, 0.6% NaCl and 0.7% disodium-hydrogencitrate in water for injection. pH was 5.0 ± 0.2 or was adjusted to pH 6.0 ± 0.2 with 1 N NaOH. Solutions were stored at 2.0–8.0 °C for a maximum of one week.
(b) [^18^F]FDG solutions were stored at 2.0–8.0 °C for a maximum of 2 days.
(c) Kry stock solutions: An appropriate amount of Kry was added to either matrix buffer pH 5.0 or pH 6.0 to give Kry concentrations of 100 mg/L Kry (pH 5.0), 1000 mg/L (pH 5.0) and 1000 mg/L (pH 6.0). These 3 stock solutions were stored at room temperature.
(d) Kry standard solutions: From the stock solutions 1000 mg/L Kry (either pH 5.0 or pH 6.0) series of standard solutions were prepared with Kry concentrations of 3.1, 6.25, 12.5, 25, 50, 75, 100 and 125 mg/L.
(e) To 900 µL of [^18^F]FDG solution 100 µL of the 1000 mg/L (pH 5.0) Kry solution were added to obtain a standard of 100 mg/L Kry in [^18^F]FDG.

Solutions (c)–(e) had a shelf life of 1 day.

### 2.4. Solutions for the Validation of Kryptofix 2.2.2 in [^18^F]FECh

As matrix buffer PBS was used (pH 7.4). When necessary, pH was adjusted to pH 6.5 using a 10% solution of NaH_2_PO_4_. Solutions of Kry in PBS or [^18^F]FECh solution were prepared similarly to the ones in the [^18^F]FDG validation, containing 100 mg/L Kry (pH 7.4 or 6.5) as well as solutions of 200 mg/L DMAE in PBS or [^18^F]FECh solution. Series of standard solutions were prepared with Kry concentrations of 3.1, 6.25, 12.5, 25, 50, 75, 100 and 125 mg/L in PBS (pH 7.4 or 6.5).

### 2.5. Solutions for the Validation of Tetrabutylammonium in [^18^F]FLT

(a) Matrix buffer: The buffer was prepared to obtain salt concentrations as in [^18^F]FLT formulations, *i.e.*, 1.10 g disodium-hydrogencitrate-1.5-hydrate, 6.28 g trisodium-citrate-2-hydrate, 3.78 g NaCl, 8.69 mL of 1 N HCl and 100 mL of ethanol (absolute) were dissolved in water for injection to give a volume of 1000 mL, resulting in pH 5.0 ± 0.2.
(b) From a stock solution of 10,000 mg/L tetrabutylammonium hydroxide 30-hydrate in matrix buffer, standard solutions were prepared with concentrations of 40, 45, 55, 70, 85, 100 and 115 mg/L of TBA.
(c) Solutions for validation of pH dependence (pH: 4.5, 5.0, 6.0, 7.0, 8.0) were prepared from solutions (b) (pH 5.0) adding either 1 N NaOH or 1 N HCl.
(d) [^18^F]FLT solutions were used as synthesized or tetrabutylammonium hydroxide 30-hydrate was added to give concentrations of 45 mg/L or 100 mg/L TBA.
(e) MeOH and NH_4_OH (25% in water) were mixed in a relation of 90:10 (v/v).

### 2.6. TLC Procedure for Analysis of Kry

Spots of 2 µL samples were applied on silica plates by means of an Eppendorf micro pipette. It proved to be mandatory that the tip slightly touched the plate and allowed the liquid to drain slowly and penetrate the silica material without any active pressure from the pipette. Plates were immediately placed on a holder inside a glass chamber for 10 min above iodine (*ca*. 5 g) covering the complete bottom of the vessel, homogenously saturating the whole chamber with iodine vapor. Plates were photographed for documentation (PowerShot SX110 IS, macro mode; jpg files, Canon, Krefeld, Germany) and visually analyzed. All experiments were *n* = 3.

### 2.7. TLC Procedure for Analysis of TBA

Spots of 2 µL samples were applied on silica plates by means of an Eppendorf micro pipette, in analogy to Kry analysis, in addition it was essential that only two spots per plate were applied. Otherwise time was to long between first and last spot application and results were invalid. Spots needed to be ca. 10 mm from the edges of the plate. After drying of spots with a cool air stream (hair dryer) 10 µL of the MeOH/NH_4_OH solution were applied on each spot. Plates were then placed in the same iodine containing chamber as for Kry, for exactly 1 min. Afterwards, plates were photographed immediately for documentation and visually analyzed (before discoloration). All experiments were *n* = 3.

## 3. Results and Discussion

TLC spot tests were validated as quality control methods to assure that residual contaminations of Kry ([^18^F]FDG, [^18^F]FECh) or TBA ([^18^F]FLT) in ^18^F-radiopharmaceutical formulations were below the limits required by e.g., Ph. Eur. Objectives of the validation experiments were specificity, accuracy and detection limit. Moreover, stability of standard solutions for routine quality control was evaluated. Validations had to be performed individually for each radiopharmaceutical, taking into account that determination of either Kry or TBA may be matrix dependent. All experiments were performed in triplicate.

### 3.1. Validation of Kry in [^18^F]FDG Formulation

Concentrations of 100 mg/L of Kry were used either in matrix or [^18^F]FDG solutions, in accordance with requirements of current Ph. Eur. [3]. The limit defined in the Pharmacopoeia is 2.2 mg/V, *i.e.*, per patient. The maximum volume in our case that could theoretically be injected in one patient is 20 mL, resulting in a calculated limit concentration of 110 mg/L. Our specification of <100 mg/L is more narrowly defined. For determination of the detection limit of the method additional solutions with concentrations from 3.1 to 125 mg/L were used. After staining with iodine, background on the plates showed an orange, light brown color. Spots of solutions containing Kry were dark brown with a diameter of 1–2 mm. In first experiments, the standard solution was prepared in WFI instead of citrate buffer. But it could be shown that as result spots were darker and of a smaller diameter. Therefore, Kry standard solutions are always prepared using buffer, to have most comparable conditions. Also, in preliminary tests it was shown that the intensity of the color of the Kry spot was dependent on the pH value of the test solution. At pH 5.0, intensity was lower than at higher pH. Therefore, buffers of standard solutions were adjusted to pH 5.0, representing the most unfavorable case. In general, content of residual Kry in [^18^F]FDG solutions (>100 productions) was far below the limit of 100 mg/L. Only in cases where pH of a solution was 6.0 or higher and the spot of the batch sample was of similar or higher intensity than the standard solution (100 mg/L; pH 5.0) there would be uncertainty. Then, the standard solution would need to be adjusted to the pH of this batch sample and the test to be repeated, to prove that Kry concentration of the batch solution was below the specified limit.

**Figure 1:**
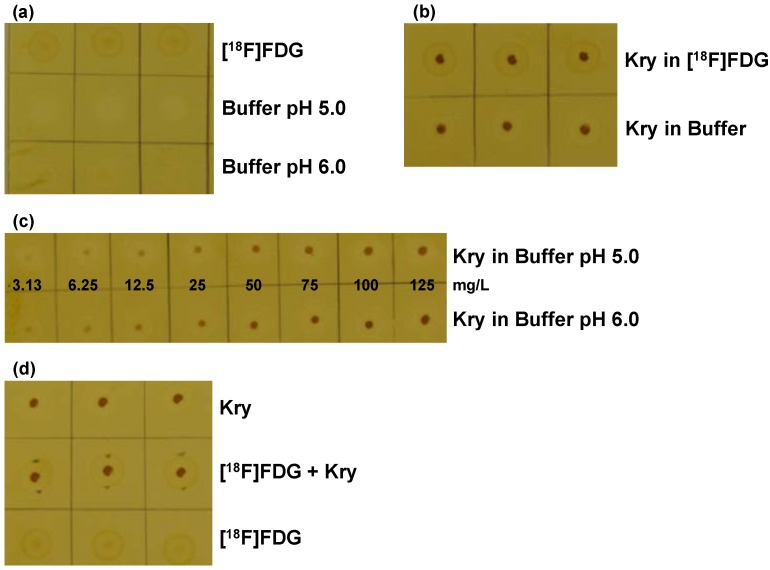
Validation of the TLC spot test for Kry in [^18^F]FDG: **(a)** Test for specificity. TLC plate after iodine staining, samples (*n* = 3) are without Kry. 1. row: [^18^F]FDG solution; 2. row: citrate matrix buffer (pH 5.0); 3. row: citrate matrix buffer (pH 6.0); **(b)** Test for Specificity. Samples (*n* = 3) of 100 mg/L Kry in either [^18^F]FDG solution (1. row) or citrate buffer matrix (pH 6.0; 2. row); **(c)** Test for specificity and detection limit. Samples of Kry standard solutions with concentrations of 3.13, 6.25, 12.5, 25, 50, 75, 100 and 125 mg/L in buffer matrix (from left to right; 1. row: pH 5.0; 2. row: pH 6.0; *n* = 3; one example shown); **(d)** Test for Accuracy. Comparison of samples (*n* = 3) of 100 mg/L Kry in matrix (pH 5.0; 1. row); [^18^F]FDG solution and 100 mg/L Kry added (pH 5.0; 2. row) and pure [^18^F]FDG solution (3. row).

To test for the specificity of the method, [^18^F]FDG solutions and plain matrix buffers (pH 5.0 and 6.0) were investigated. With matrix buffers no coloration of the spots was visible, with [^18^F]FDG a very weak, diffuse, light brown spot developed that was completely different from Kry spots from standard solutions (Figure 1a,b). Comparison of standard solutions of 100 mg/L of Kry in either [^18^F]FDG solution or matrix buffer (pH 6.0) showed small, dark spots of comparable intensity (Figure 1b). Third, the two series of standard solutions (pH 5.0 and pH 6.0) with Kry concentrations of 3.1, 6.25, 12.5, 25, 50, 75, 100 and 125 mg/L were investigated (Figure 1c). Within both series spots differing in concentrations by a factor of 2 could be distinguished. As already shown in the preliminary tests, spots from a certain concentration at pH 6.0 corresponded to spots from the series at pH 5.0 with twice the concentration.

From the same set of experiments the detection limit of the method was identified to be 6.25 mg/L, from a clearly visible spot. For proving the accuracy of the method, three types of spots were compared (Figure 1d): Kry in buffer (100 mg/L; spot 1), spot 2 was from 2 µL of [^18^F]FDG solution, after drying for 10 s with a hair dryer 2 µL of Kry in buffer (100 mg/L) were added on top and spot 3 was from pure [^18^F]FDG solution. On one TLC plate buffer of pH 5.0 was used (spots 1 and 2), on a second plate buffer of pH 6.0 was applied. Independent from pH value spots 3 were hardly visible, whereas spots 1 and 2 showed same shape and intensity. The results from these validations met the defined specifications from the respective validation plan, proving the applicability of the described TLC method as test for residual Kry in [^18^F]FDG formulations, regarding specificity, accuracy and detection limit.

For routine quality control it was decided to use a standard solution containing 100 mg/L of Kry in matrix buffer pH 5.0, which then is tested against the individual [^18^F]FDG batch. This standard solution is prepared as large batch and stored in portions of 1 mL in small glass vials at <−15 °C. In frame of the validation the stability of the standard solution was evaluated after 2 weeks, 1 month, 3 months and 6 months by comparison with a freshly mixed solution. 3 different batches of standard solution were prepared. These stability studies demonstrated that spots from stored solutions showed no difference to spots from fresh solutions. Consequently, the shelf life of the Kry standard solution was specified to be 6 months.

### 3.2. Validation of Kry in [^18^F]FECh Formulation

Validation was performed in a similar way as for [^18^F]FDG, regarding specificity, accuracy, detection limit and stability of standard solutions. Kry standard solutions had the same concentration of 100 mg/L as limit, as there is no monograph for [^18^F]FECh yet available. Calculating from our batch volume of 13 mL that might be injected in a patient, a limit concentration of 169 mg/L would result (2.2 mg/V). Like for [^18^F]FDG, our limit is narrower. Besides the difference in matrix buffer ([^18^F]FDG: citrate, ethanol as stabilizer *vs.* [^18^F]FECh: PBS), [^18^F]FECh formulations may also contain residual DMAE (specified limit: 200 mg/L), which may interfere with the test and indeed preliminary experiments showed light brownish coloration of spots from DMAE. Secondly, as in the [^18^F]FDG validation a pH dependence of the test was shown (lower pH values resulted in spots of a less intense color) and here, the specifications for [^18^F]FECh are pH 6.5–8.5 (PBS has a pH of 7.4) the same effect might occur here as well. Consequently, in a comparative study solutions at pH of 6.5 and pH 7.4 needed to be validated as well.

**Figure 2:**
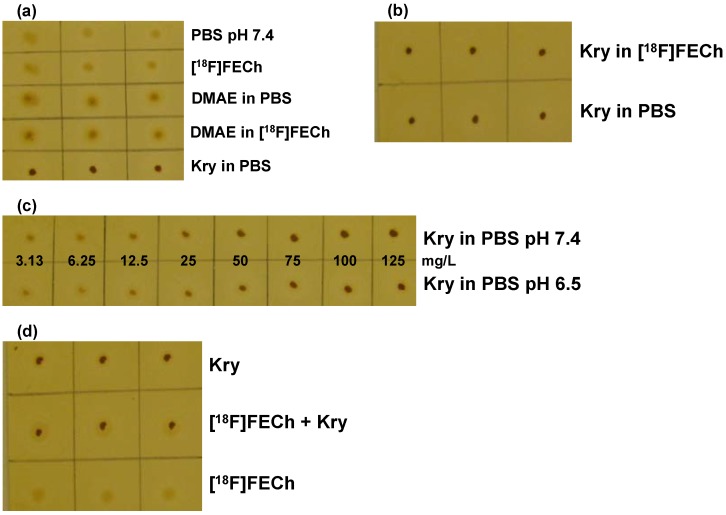
Validation of the TLC spot test for Kry in [^18^F]FECh: (**a**) Test for specificity. 1. row: PBS buffer (pH 7.4); 2. row: [^18^F]FECh solution; 3. row: 200 mg/L DMAE in PBS; 4. row: 200 mg/L DMAE in [^18^F]FECh solution; 5. row: 100 mg/L Kry in PBS (*n* = 3); (**b**) Test for specificity. Samples (*n* = 3) of 100 mg/L Kry in either [^18^F]FECh solution (1. row) or PBS (2. row); (**c**) Test for specificity and detection limit. Samples of Kry standard solutions with concentrations of 3.13, 6.25, 12.5, 25, 50, 75, 100 and 125 mg/L in PBS (from left to right; 1. row: pH 7.4; 2. row: pH 6.5; *n* = 3; one example shown); **(d)** Test for Accuracy. Comparison of samples (*n* = 3) of 100 mg/L Kry in PBS (pH 7.4; 1. row); [^18^F]FECh solution with 100 mg/L Kry added (pH 7.4; 2. row) and pure [^18^F]FECh solution (3. row).

In general, coloration of plates and spots was similar to the [^18^F]FDG validation. Specificity of the test was proven comparing matrix buffer, [^18^F]FECh solution, 200 mg/L DMAE in buffer and 200 mg/L DMAE in [^18^F]FECh solution *vs.* 100 mg/L Kry in matrix buffer standard solution. All spots from samples without Kry were much less intensive (Figure 2a), demonstrating especially that the disturbing effect of DMAE is negligible. Secondly, spots from solutions of 100 mg/L Kry in matrix buffer or [^18^F]FECh solution were dark brown and of same intensity (Figure 2b). Thirdly, using the solutions from the second experiment, but adjusted to pH 6.5 gave again for 100 mg/L Kry in buffer or [^18^F]FECh solution, spots of same intensity (plates not shown). And finally, two series of standard solutions (pH 6.5 and 7.4) with Kry concentrations of 3.1, 6.25, 12.5, 25, 50, 75, 100 and 125 mg/L in PBS were investigated (Figure 2c). In both series differences in concentration by a factor of 2 were clearly visible. Intensities of spots were similar for same concentrations, showing that at least at the investigated pH values no pH dependence of the test could be observed. pH 8.5 (upper specification limit of [^18^F]FECh formulation) was not investigated, as (if at all) spots should be more intensive at higher pH (see [^18^F]FDG), resulting in a false result of a Kry concentration higher than the real value, which is uncritical from a pharmaceutical point of view.

For defining the detection limit the series of Kry concentrations (Figure 2c) were analyzed. The spot from the Kry concentration of 12.5 mg/L was clearly visible, detection limit was therefore well below the specified nominal value of “≤50 mg/L”. Compared to the [^18^F]FDG validation, detection limit was higher for Kry in [^18^F]FECh solutions.

Accuracy of the method was again proven by comparing spots of 100 mg/L Kry in matrix buffer (1); [^18^F]FECh solution, drying, addition of 100 mg/L Kry in buffer (2) and as spot 3 [^18^F]FECh solution was applied. Experiments were again performed at pH 6.5 and 7.4, showing no pH dependence. In both cases spots 3 showed minimum coloration, spots 1 and 2 were of same intensity (Figure 2d). Overall, validation was performed successfully, clearly demonstrating the feasibility of this TLC spot test.

Stability of the standard solution to be used in routine quality control (100 mg/L Kry in PBS matrix buffer, pH 7.4), stored in 1 mL portions at <−15 °C was proven to be 6 months (three different batches; performance as in [^18^F]FDG validation).

### 3.3. Validation of TBA in [^18^F]FLT Formulation

After the successful application of the TLC spot test for detection of Kry in [^18^F]FDG or [^18^F]FECh solution, now it was investigated, if this test was also valid for detection of TBA in [^18^F]FLT formulation. From first tests it was obvious that before staining with iodine, 10 µL of MeOH/NH_4_OH (90:10 *v/v*) must be applied on each spot to detect TBA and development times in the iodine chamber had to be 1 min. [^18^F]FLT solution was a citrate buffer like [^18^F]FDG formulation, but containing ca. 10% of ethanol (*v/v*) in contrast to [^18^F]FDG with only *ca*. 0.3% of ethanol (*v/v*). Preliminary experiments showed no influence of ethanol content on the test. Again, pH dependence needed to be tested, as the TBA standard solution was planned to have a pH of 5.0, specification for [^18^F]FLT formulation is pH 4.5–8.0.

**Figure 3:**
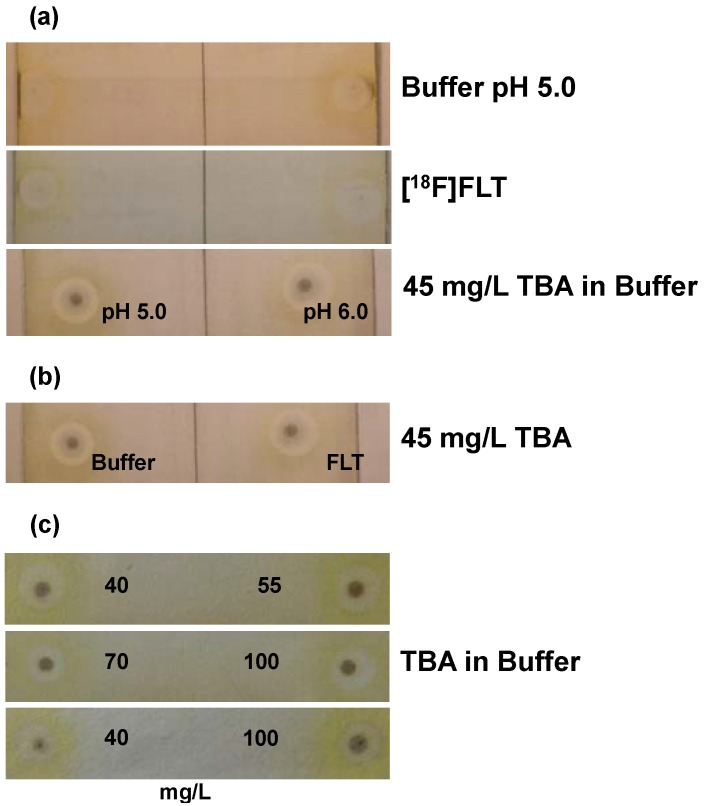
Validation of the TLC spot test for TBA in [^18^F]FLT: **(a)** Test for specificity (2 spots per plate; *n* = 3; one example shown). Upper plate: citrate buffer matrix (pH 5.0); middle plate: [^18^F]FLT solution; lower plate: 45 mg/L TBA (left pH 5.0; right pH 6.0); **(b)** Test for specificity. Samples (example shown) of 45 mg/L TBA in either citrate buffer matrix (pH 5.0; left) or [^18^F]FLT solution (right); **(c)** Test for specificity and detection limit. Examples for the comparison of samples of TBA standard solutions with concentrations of 40, 55, 70 and 100 mg/L in citrate buffer matrix (pH 5.0).

To meet Ph. Eur. requirements for the limit of TBA impurities in ^18^F-radiopharmaceuticals (2.6 mg/V) the general standard solution contained 100 mg/L TBA, as the maximum volume in our case is 21 mL, *i.e.*, the calculated limit from Ph. Eur. would be 124 mg/L. For determination of the detection limit and to see what differences in concentrations could be differentiated, series of solutions were prepared with TBA concentrations of 40, 45, 55, 70, 85, 100 and 115 mg/L. After iodine staining, plates showed a yellow-grey color, spots of solutions containing TBA were dark brown (diameter of 1–2 mm surrounded by grey concentric circles). For validation of specificity matrix buffer solutions (pH 5.0 and pH 6.0), [^18^F]FLT solutions and standard solutions containing 45 mg/L TBA (pH 5.0 and pH 6.0) were compared. Spots from matrix gave almost no coloration as well as [^18^F]FLT solutions; but from standard solutions spots were clearly visible (Figure 3a). No pH dependence was observed. Standard solutions containing 45 mg/L TBA and [^18^F]FLT solutions containing 45 mg/L TBA gave spots of same intensity (Figure 3b). Comparison of spots from the series of concentrations showed that only differences in concentrations of ≥60 mg/L could be visualized definitely and also that only spots on the same plate were comparable due to plate individual results of staining (Figure 3c). The spot from the standard solution of 40 mg/L of TBA was clearly visible and therefore this concentration was defined as detection limit (Figure 3c). Accuracy of the method could be demonstrated by comparing spots from TBA solutions (45 mg/L), spots from [^18^F]FLT solutions (pure) and spots from [^18^F]FLT solutions with TBA standard (45 mg/L) added. This set of experiments was performed with solutions at pH 5.0 and pH 6.0. No pH dependence was visible. Spots from [^18^F]FLT solutions were hardly visible. Spots from solutions containing TBA were clearly visible and of same intensity (see examples in Figure 3a–c. To check for the pH dependence over the whole range of specification, standard solutions with 100 mg/L of TBA and pH 4.5, 5.0, 6.0, 7.0, 8.0 were tested. No difference in intensity was visible at any pH value (plates not shown). These results demonstrated the applicability of the TLC spot test also for TBA in [^18^F]FLT formulation. In analogy to [^18^F]FDG and [^18^F]FECh validations, stability of the standard solution for routine quality control (100 mg/L TBA in matrix buffer, pH 5.0), stored in 1mL portions at <−15 °C was verified so far for up to 1 month.

### 3.4. General Discussion

Overall, the presented and validated TLC spot test proved its capability for determination of residual Kry in [^18^F]FDG and [^18^F]FECh formulations with the perspective to become a general method. Indeed, validations have to be carried out for each tracer formulation and its individual matrix, as organic impurities (e.g., amines) might give false positive or stabilizers false negative results. So far, our results never showed disturbing interferences of major concern. No problems arose from neither ethanol nor residual DMAE (in [^18^F]FECh solutions) or pH dependencies of spot intensities. Latter effect would lead to the necessity of pH adjustments of standard solutions for valid results of each batch in daily routine, but on the one hand residual Kry and also TBA concentrations close to the specified limits were never observed, normally they are not detectable—and detection limits are low. On the other hand, in the investigated radiopharmaceuticals (all in buffered solutions) alterations in pH values were minimal (Table 1).

**Table 1:** Compilation of parameters investigated and results in the described validations regarding specificity, accuracy and detection limit of the TLC spot test for residual Kry in [^18^F]FDG and [^18^F]FECh as well as TBA in [^18^F]FLT.

Parameters	Kry in FDG	Kry in FECh	TBA in FLT
Nominal limit/mg/V ^1^ mg/L ^2^	<2.2 <100	<2.2 <100	<2.6 <100
*Specificity*			
Color of spots for:			
Product solution	minimal	minimal	minimal
Matrix buffer	minimal	minimal	minimal
100 mg/L reagent solution	clearly visible	clearly visible	clearly visible
Intensity of spots of reagent in buffer or product solution comparable	YES	YES	YES
Distinguishability of spots with concentration difference of	Factor 2	Factor 2	≥60 mg/L
pH dependence	not relevant	NO	NO
Effect of ethanol	NO (0.3%) ^3^	n. a.	NO (10%) ^3^
Effect of DMAE	n. a.	minimal	n. a.
*Accuracy*			
Addition of reagent to product gives comparable spots to reagent in buffer	YES	YES	YES
*Detection limit*/mg/L	6.25	12.5	40
Reagent in matrix standard used in routine	100 mg/L pH 5.0	100 mg/L pH 7.4	100 mg/L pH 5.0
Stability (months at <−15°C)	6	6	1

^1^ Ph. Eur.; ^2^ our institution; ^3^ ethanol concentration in brackets; n. a. = not applicable.

Whereas the test for Kry proved its robustness, the test for TBA reacted more sensitively to variations and instructions for performance of the test had to be followed strictly, like fast administration on plate, drying, time of development *etc*. Only two spots could be administered on one plate, to minimize time before iodine staining, otherwise results were erroneous. Only spots on one plate were comparable, which made validation laborious, but gives no problem in routine analysis, when only sample and standard solution are applied. Detection limit was higher for TBA than for Kry and concentrations of TBA spots that were to be compared needed to differ by 60 mg/L to give valid results. Still, these aspects are no problem for routine analysis, as TBA concentrations in [^18^F]FLT formulations from routine production were found to be very low (see Figure 3a), and then determination of a concentration of “<100 mg/L” is reliably assured (Table 1).

This TLC spot test is designed to determine required limits to be met and not to absolutely quantify the concentration of Kry or TBA present, although this was possible for [^18^F]FDG or [^18^F]FECh. Nevertheless, in routine quality control for chemical impurities the specification “below limit” (e.g., “<100 mg/L”) is absolutely sufficient and in conformity with Pharmacopoeia norms.

## 4. Conclusions

A TLC spot test for determination of residual Kry or TBA in ^18^F-radiopharmaceutical formulations was successfully developed and formally validated for specificity, accuracy and detection limit for [^18^F]FDG, [^18^F]FECh (Kry) and [^18^F]FLT (TBA). Interference with various matrix effects was negligible; the test is adequate, easy to handle, very fast and resources saving (no expensive reagents or HPLC system necessary). Now, it is already implemented in routine quality control of these three tracers in our institution, in case of [^18^F]FDG it was formally accepted by authorities within our marketing license. With these positive results, the test is now under validation for further ^18^F-radiopharmaceuticals.
